# Epigenetic Effects of n-3 LCPUFAs: A Role in Pediatric Metabolic Syndrome

**DOI:** 10.3390/ijms20092118

**Published:** 2019-04-29

**Authors:** Matilde Amatruda, Giulio Ippolito, Sara Vizzuso, Giulia Vizzari, Giuseppe Banderali, Elvira Verduci

**Affiliations:** Department of Pediatrics, San Paolo Hospital, University of Milan, Via A. Di Rudinì 8, I - 20142 Milan, Italy; Giulio.ippolito@unimi.it (G.I.); Sara.vizzuso@unimi.it (S.V.); Giulia.Vizzari@unimi.it (G.V.); Giuseppe.banderali@unimi.it (G.B.); Elvira.verduci@unimi.it (E.V.);

**Keywords:** DHA, LCPUFAs, pediatric obesity, metabolic syndrome, epigenetics, nutritional programming

## Abstract

Childhood obesity represents an important public health issue worldwide and is strongly linked to metabolic alterations such as hypertension, insulin resistance, and dyslipidemia. The constellation of these conditions is commonly known as Metabolic Syndrome (MetS). Metabolic syndrome is not just a simple cluster of metabolic complications due to excess of adipose tissue, but is considered a risk factor for cardiovascular diseases. Evidence from several human and animal studies suggests that environmental and nutritional exposure during pregnancy may affect the newborn development and future health through epigenetic changes, playing a potential role in determining obesity and obesity-related complications. Understanding how nutritional epigenetic mechanisms contribute to the “transgenerational risk” for obesity and metabolic dysfunction is crucial in order to develop early prevention strategies for children’s health. Nutrigenetics is the science that studies the role of nutrients in gene expression. Long Chain Polyunsaturated Fatty Acids (LCPUFAs) are known for their health benefits, especially in relation to their ability to modulate inflammation and improve some obesity-associated comorbidities, mainly by decreasing plasma triglycerides. Recent nutrigenetic research is focusing on the potential role of LCPUFAs in influencing epigenetic markers. In this review, we present the most recent updates about the possible interaction between n-3 LCPUFAs and epigenetic pathways in metabolic syndrome. Literature from MEDLINE^®^ and the Cochrane database between May 2005 and December 2018 has been scanned.

## 1. Introduction

Childhood obesity is an important public health problem all over the world. The prevalence of obesity has increased in school-aged children from the 1980s (from 6.5% to 18% in children and from 5.0% to 18.4% in adolescents) to the present [1,2]. From recent epidemiologic studies emerges a ‘’plateau trend’’ showing that, in the last few years, the percentage of overweight and obese children/adolescents remained almost stable in both sex groups [3]. Currently, one out of three children in the United States is overweight or obese [4].

The WHO European Childhood Obesity Surveillance Initiative (COSI), in its third edition in 2012, revealed that overweight and obesity rates among primary school-children ranged from 18–52% in boys and from 13–43% in girls. The data showed a higher percentage of obesity in Southern European countries, although these are traditionally recognized as models of the Mediterranean diet [5]. 

According to the Italian Government surveillance project “Okkio alla salute”, 21.3% of primary school children were overweight and 9.3% obese. Between the years 2008 and 2016, the prevalence of obesity decreased from 12% to 9.3% (with a percentage reduction of about 22%), while overweight prevalence decreased from 23.2% to 21.3% (with a percentage reduction of about 8%). 

Globally, in less than 10 years, adiposity excess in children has decreased about 13%, from 35.2% in 2008/2009 to 30.6% in 2016 [6]. Despite this ‘’promising’’ trend, pediatric obesity’s spread is still high [5,6]. 

Facing pediatric obesity, it is crucial not just for its organic and psychosocial implications in adulthood: indeed, clinical issues and preclinical alterations can be found also in children [7].

A chronic, low-grade inflammatory state driven by obesity leads to metabolic alterations responsible for vascular dysfunction, thrombotic disorders, and multiple organ damage: this metabolic dysfunction could determine clinical conditions such as hypertension, dyslipidemia, and insulin resistance [8]. Metabolic Syndrome (MetS) is a cluster of clinical disorders associated with a higher cardiovascular risk: according to the most recent meta-analyses, cardiovascular outcome and all-cause mortality are higher in patients with metabolic syndrome [9]. Though genetic, socio-economic, and environmental factors have been cited as possible MetS drivers, unhealthy eating habits play a major role. The “Westernized diet”, characterized by high protein content, saturated fats, refined grains, sugar, and salty foods with reduced consumption of fruits, vegetables, and polyunsaturated fat sources, is a well-known risk factor for cardio-metabolic complications and the incidence of metabolic syndrome [9]. 

Hence, strategies for prevention and treatment of pediatric obesity are needed to reduce short- and long-term morbidities and limit their impact on healthcare system costs [10].

Is has recently emerged that epigenetic modifications could play a role in the development of obesity and metabolic syndrome. Epigenetics include all heritable changes in the regulation of gene activity, not altering the DNA sequence. Several human and animal studies’ results suggest that environmental factors influence fetal development, determining metabolic pathways that, if persistent, could lead to obesity in the newborn.

This “gestational programming”, triggered by suboptimal maternal nutrition/endocrine factors, seems to play a role mainly through epigenetic modifications rather than changes in the DNA sequence. DNA methylation, histone modifications, and small non-coding microRNAs constitute the three basic mechanisms of epigenetic changes. All of these have been brought into play as possible factors in modulating gene expression and promoting metabolic syndrome features [11].

Due to the potential reversible nature of epigenetic changes, disclosing their contribution to transgenerational transmission of obesity and metabolic dysfunction is crucial to formulate detection and prevention strategies to avoid “programmed” metabolic syndrome.

LCPUFAs are well known for their health benefits, especially in relation to their ability to modulate inflammation and improve some obesity-associated complications such as insulin resistance, hypertension, and dyslipidemia by decreasing plasma triglycerides. Recent research is focusing on their potential to change epigenetic markers.

## 2. Topic of Review

While the health benefits of LCPUFAs are widely studied and recognized, it is a matter of debate whether these effects may be explained by epigenetic processes. 

The aim of this paper is to discuss, according to current knowledge, the presumed epigenetic mechanisms of n-3 LCPUFAs and their potential role in metabolic syndrome components.

## 3. Biochemistry and Biologic Function of LCPUFA

LCPUFAs are fatty acids of 18–20 carbons or more, assembled in two main families according to the first double bond position from the methyl end group: ω6 (n-6) and ω3 (n-3). The main n-3 family LCPUFAs are α-Linolenic Acid (ALA) (18:3 Δ9, 12, 15), Eicosapentaenoic Acid (EPA) (20:5 Δ5, 8, 11, 14, 17), Docosapentaenoic Acid (DPA) (22:5 Δ7, 10, 13, 16, 19), and Docosahexaenoic Acid (DHA) (22:6 Δ4, 7, 10, 13, 16, 19) (Table 1). The n-6 LCPUFA family includes Linoleic Acid (LA) (18:2 Δ9, 12) and Arachidonic Acid (ARA) (20:4 Δ5, 8, 11, 14) (Table 2).

The LCPUFAs are synthesized in the liver endoplasmic reticulum and peroxisomes through desaturase and elongase enzymes. EPA and DHA are absorbed from the gastrointestinal tract and transported to the liver through triglycerides in chylomicron particles. From the liver, they are brought into blood circulation in lipoproteins such as Low-Density Lipoprotein (LDL) cholesterol, High-Density Lipoprotein (HDL) cholesterol, and as plasma phospholipids. In a smaller proportion, they are present as free fatty acids bound to albumin. EPA and DHA are converted into cell membrane phospholipids, especially in the heart, brain, and in adipose tissue as triglycerides.

DHA is the biologically active end product of alpha-linolenic acid, which is an essential fatty acid because it is exclusively obtained from the diet. ALA is referred to as the essential precursor of n-3 LCPUFAs, while Linoleic Acid (LA) gives rise to n-6 LCPUFAs. ALA is converted into long chain fatty acids by a series of alternating desaturations and elongations. The first step in ALA metabolism is desaturation, catalyzed by delta-6-desaturase. This step is considered rate-limiting. Since the conversion efficiency of ALA to Docosahexaenoic Acid (DHA) and Eicosatetraenoic Acid (EPA) is low and limited in humans, looking for dietary sources is essential to achieve adequate provision of DHA. DHA is primarily derived from fish, especially oily fish such as herring, salmon, and tuna, as well as omega-3-fed chickens and their eggs. Eicosapentaenoic Acid (EPA) is a precursor of DHA and is metabolized to DHA in the liver [12].

The intake of LCPUFAs, particularly of EPA and DHA, in the past few years has been encouraged mostly through seafood consumption. 

Strobel and colleagues determined the n-3/n-6 fatty acids’ ratio, including the EPA and DHA amounts, in fish products (123) from the German market. They found that the content of LCPUFAs varies among the fish species, according to fat composition and type of feeding. Therefore, according to these results, a variety of different fish species should be consumed [13].

In recent years, the risks related to the consumption of seafood and fish oils in particular areas have been highlighted [14]. Fatty fish and large predators are at a major risk of contamination with environmental pollutants and heavy metals, as hydrophobic substances accumulate in lipid depots. This may be dangerous especially for women during pregnancy and breast feeding and young children [15]. 

Recent knowledge has stimulated research into alternative sources of n-3 LCPUFAs in different fields, and microalgae are emerging as a promising, sustainable resource for LCPUFAs’ production, as a replacement for fish oil [16]. However, from the majority of experimental and epidemiological studies, it turned out that the benefits of fish intake go beyond the potential risks, even for vulnerable groups [17].

Several animal studies, observational studies, and clinical trials evaluated the health properties of n-3 LPUFAs, concluding that EPA and DHA are the most active components [18]. 

These FAs influence diverse homeostatic pathways also through derived bioactive products, such as eicosanoids. Pro-inflammatory eicosanoids are originated from ARA, while EPA and DHA give rise to anti-inflammatory eicosanoids. Thus, a higher consumption of n-3 PUFAs has a beneficial role against a large spectrum of diseases that share a chronic inflammatory trigger [19].

Attention to these nutrients was first given when a lower incidence of Cardiovascular Diseases (CVD) was found in populations with regular fish consumption in their diet (e.g., Inuits), in comparison to others consuming less seafood and more n-6 PUFA [20]. 

The importance of LCPUFA and their nutritional value for human health is clearly evident. Evidence shows that a diet rich in palmitic acid, a saturated fatty acid, is correlated with lower insulin sensitivity and increased risk of atherosclerosis. By contrast, a high intake of EPA and DHA has beneficial effects, especially lowering triglyceride levels and increasing HDL levels [21].

## 4. Metabolic Syndrome in Children

Children who are overweight and pediatric obesity represent an emerging public health priority as rates have rapidly increased worldwide. Obesity is often associated with other metabolic abnormalities, known globally as Metabolic Syndrome (MetS). In 2005, the International Diabetes Federation (IDF) formulated its definition of MetS in adults [22]. Although this combination of metabolic disorders was thought to be an adult-onset disease, MetS prevalence is also increasing in children and adolescents [23].

In recent years, it has become evident that the adult definition could not be applied to children because of a variety of evolving conditions during growth development. In 2007, meeting the urgency to make early diagnosis and prevention strategies in the pediatric population, the IDF suggested a consensus definition of MetS in children and adolescents. The IDF definition is different according to age-groups: age 6sixyears to younger than 10 years; age 10 years to younger than 16 years; and 16 years or older (children younger than six years were not included due to insufficient data available). In all groups, abdominal obesity is the necessary condition to make the diagnosis. The IDF consensus also recommends not to diagnose metabolic syndrome in children younger than 10 years; while it is strongly suggested to focus all efforts on weight reduction in this age group. 

For the group of children aged 10 years or older, metabolic syndrome can be diagnosed with abdominal obesity and associated with two or more other clinical features, among which are high blood pressure, elevated triglycerides, low HDL-cholesterol, and increased plasma glucose. For children older than 16 years, IDF suggests to use adult criteria [24].

## 5. Impact of LCPUFA on MetS Features during Lifetime

### 5.1. Evidence in Adults

Metabolic syndrome is a constellation of features that increase a patients’ cardiovascular risk. Several definitions for metabolic syndrome in adults have been proposed, leading to some difficulty in comparing data from studies using different criteria [25]. The 2001 National Cholesterol Education Program (NCEP) Adult Treatment Panel III (ATP III) [26] (last update in 2005) and the International Diabetes Federation (IDF) 2006 criteria are the most widely used [22]. All proposals agree on the basic components of metabolic syndrome: obesity, dyslipidemia, insulin resistance, and hypertension. 

It is currently accepted that supplementation with n-3 LCPUFAs might improve some metabolic syndrome features such as insulin resistance, hypertension, and dyslipidemia, mainly by decreasing plasma triglycerides. Several meta-analyses have confirmed that the most consistent action of ω3 LCPUFAs is the reduction in triglycerides [27]. 

Serum triglyceride levels can be lowered up to 25–30 percent through fish oil consumption. Little triglyceride lowering is seen with dietary doses or low-dose (<1 g/day) supplementation, whereas higher doses (3–4 g/day) result in appreciably lower triglyceride levels. According to a meta-analysis of 55 placebo-controlled trials, each 1 g/day increase in EPA + DHA supplementation decreases triglycerides by 5.9 mg/dL (0.07 mmol/L) [28]. 

A recent review of 17 RCT confirmed that the continuative administration of n-3 LCPUFAs doses >1 g for three months has a beneficial effect also in metabolic syndrome patients, determining a reduction in triglycerides from 7–25% [29].

A decreased transport of free fatty acids to the liver seems to better explain how fish oil lowers triglyceride concentration: this is obtained through enhancement of lipoprotein lipase-mediated extracellular lipolysis in different tissues (adipose, heart, and muscle) and hepatic/skeletal muscle β-oxidation. Fish oil also plays a role in reducing intracellular lipolysis by suppression of inflammation in adipose tissue [30].

Fish oil supplementation seems to have a beneficial secondary effect on blood lipid profile also by modestly raising HDL cholesterol concentrations and lowering the proportion of small dense LDL cholesterol, VLDL, and chylomicron particles [31].

Fish oil supplementation has little effect on systolic and diastolic Blood Pressure (BP), as documented by a meta-analysis of 70 randomized controlled trials: consumption of DHA + EPA leads to a reduction of systolic and diastolic BP values by 1.52 and 0.99 mmHg, respectively [31]. The effect is even more evident in hypertensive patients not in pharmacological treatment than in those who are normotensive. Consumption of more than a 2 g/die dose leads to a more significant reduction in diastolic blood pressure [32]. 

Furthermore, average BP levels at 24 h of ambulatory monitoring are decreased by omega 3 consumption [33]. 

The anti-hypertensive properties of fish oil are due to improvement in endothelial function, reduction of vascular resistance, and modulation of cardiac output [34,35]. These effects are mediated by eicosanoids epoxides (derived from DHA and EPA) with increased production of nitric oxide (vasodilator agent) and reduction in markers of endothelial dysfunction (E-selectin, VCAM1, ICAM1) and proinflammatory cytokines (IL1 β and TNF α) [36,37].

In meta-analyses of randomized trials, biomarkers of glucose metabolism or insulin sensitivity in patients with or without diabetes seem not to be affected by n-3 PUFAs [38]. According to a meta-analysis of 18 prospective cohort studies including 540.184 participants and 25.670 cases of incident type 2 diabetes, there is no significant association between incidence of diabetes and fish/seafood consumption or estimated intake of EPA plus DHA [39]. Our study group has recently focused the attention on Peroxisome-Proliferator-Activated Receptor-gamma (PPARγ2) [40]. PPAR is a family of proteins that act as transcriptional factors, belonging to the ligand-activated nuclear receptor superfamily: three isoforms of PPAR are known (PPAR-α, PPAR-β/δ, and PPAR-γ). Among these, the alfa and gamma isoforms are involved in glucose and lipid homeostasis and insulin sensitivity [41]. PUFAs and derivatives, such as arachidonic and docosahexaenoic acid, seem to activate PPAR gamma, which leads to an increased insulin sensitivity [42]. 

PPAR receptors are well-recognized therapeutic targets for diabetes, but emergent studies state that their modulation could act as a therapeutic strategy also for metabolic syndrome. 

### 5.2. Trials in Children

Despite the extensive literature available for adults, few clinical trials have been performed to study if DHA supplementation has an effect on metabolic syndrome determinants in children. There is emerging evidence from epidemiological and clinical studies that an increased intake of omega 3 fatty acids could affect the degree of obesity, reducing body fat mass and enhancing weight loss in adults [43]. 

Consistent with this, it has been demonstrated that lower levels of n-3 fatty acids are associated with increased body weight in children, but there is no clear evidence that n-3 supplementation has an impact on adiposity or BMI [44]. A recent double-blind trial evaluated the effect of daily fish oil supplementation (1.2 g of n-3 LCPUFAs) vs. placebo (sunflower oil) in a cohort of 366 obese adolescents following a hypocaloric diet: no difference in body weight, HOMA index, and insulin level changes were found between the two groups [45]. Another randomized double-blind trial studied the effects of 15 weeks of supplementation of n-3 fatty acids vs. placebo (rapeseed oil) in 423 children aged 8–9 years old, measuring body weight and Physical Activity (PA). No significant difference in neither PA nor BMI between the two groups was found [46]. 

Nobili and colleagues evaluated the impact of DHA supplementation at low or high doses (250–500 mg/die) for six months vs. placebo on hepatic steatosis detected by ultrasonography, in a cohort of 60 children with NAFLD. DHA supplementation, in a non-dependent dose manner, resulted in less severe hepatic disease, reduction of triglycerides, and an increase in insulin sensitivity, but had no effect, neither on alanine transaminase levels nor body mass index [47]. In contrast with this finding, another study reported that in 201 obese and insulin-resistant children and adolescents who were randomized to receive 500 mg of metformin or 1.8 g of n-3 PUFAs for 12 weeks, n-3 FAs decreased glucose and insulin levels (reducing HOMA-IR values and also having a meaningful impact on BMI [48]. 

Another clinical trial was performed to evaluate the levels of adipokine in 76 overweight children and adolescents with insulin resistance, not on diet, who took 900 mg of n-3 LCPUFA supplement or placebo for one month. After treatment, insulin and HOMA-IR were significantly decreased in the intervention group, which showed also lower values of serum adiponectin, leptin, and TNF alfa after treatment. Despite no differences at baseline, only the n-3-FAs group had decreased fasting insulin and HOMA-IR. Interaction between supplementation and weight loss was found to be significant [49].

One double-blind placebo randomized trial recruited 25 pediatric patients with hypertriglyceridemia. Values of TG ranged from 150–1000 mg/dL in the study population, who was given a high daily dose of DHA + EPA (3360 mg/die) vs. placebo for six months. There was no significant difference between the two groups in lowering TG levels: similar results were reached by previous studies, which found minimal or no effect of supplementation on TG levels. Statistical analysis showed no difference in weight loss, insulin resistance markers, HDL cholesterol, BMI, waist circumference, and systolic blood pressure, suggesting that LCPUFA supplementation could not influence the prevalence of metabolic syndrome [50].

A study evaluated the effect of LCPUFA at high doses on subcutaneous adipose tissue and blood analysis in 26 obese adolescents: five daily doses of EPA + DHA (3 g per day of 944 mg EPA and 2088 mg DHA) for 12 weeks resulted in a reduction of BMI, waist circumference, and serum triglycerides values. Analysis of the subcutaneous adipose tissue through biopsy suggested a LCPUFAs-mediated gene modulation: PPAR-γ and PGC-1a were downregulated, while PPAR-α and SREBP1 were upregulated [51]. These results agree with the literature, which states that PPAR-α is responsible for lipid homeostasis and thereby prevents dyslipidemia [52]. PPAR-γ is involved in adipocyte differentiation and glucose homeostasis: it improves insulin sensitivity by opposing TNF-α activity in adipocytes [53]. For this purpose, PPAR-receptor agonists are used for their hypoglycemic properties as a therapeutic strategy of diabetes in adults.

In sum, n-3 LCPUFAs have demonstrated anti-inflammatory and metabolic properties, widely accepted in the adult population, but their potential therapeutic role in children who are obese and have metabolic alterations is still debated. Well-structured and long-term clinical trials are needed to verify their beneficial role in children health. 

### 5.3. Supplementation in Pregnancy and Early Life 

The “gestational programming” hypothesis explains how maternal nutritional status, hormonal balance, and metabolic environment affect gene expression and ultimately metabolism of the offspring [54]. Support for the addition of LCPUFAs in pregnant mothers or infant formula is based on recent research that demonstrated n-3 LCPUFAs’ importance in the developing brain and retina during the last trimester of pregnancy up to the second year of life [55,56]. On the other hand, few studies have focused attention on the potential benefits of LCPUFAs on preventing newborn risk of obesity and its metabolic complications. It is unclear how LCPUFA supplementation during pregnancy can affect offspring development and metabolism: numerous clinical trials have been performed with conflicting results.

A double-blind randomized controlled trial involving 350 women who consumed an algal oil source of DHA 600 mg/die vs. placebo from <20 week of gestation to birth led to a higher duration of pregnancy and infant biometry, not affecting the ponderal index, intended as the proportion of fat and lean mass. Fish oil could be responsible for longer pregnancy due to the effect of EPA and ARA-derived prostaglandins [57].

To some extent, LCPUFAs may have a role in preventing preterm delivery and LBW, which could represent an important clinical outcome in relation to the increased risk of accelerated weight gain and obesity in these categories [58]. 

The Domino trial was a controlled randomized study in which pregnant women took high doses of DHA vs. placebo from the 20 th week of gestation until delivery; a subsequent study with a seven-year follow-up was conducted among 252 sons and daughters of the enrolled mothers. Between the two groups, there was no difference in biometrics, neither in body composition assessed by displacement plethysmography (BOD POD) and Bioelectrical Impedance Spectroscopy (BIS). The results were in accord with the previous reports made at three and five years of follow-up of the same cohort [59].

A clinical trial of DHA supplementation has been performed also in the risk categories of pregnant women. 

A study led by Foster and colleagues studied the effects of DHA 800 mg/day supplementation vs. placebo in obese or diabetic pregnant women from 25–29 weeks of gestation. Differences of adiposity were not significant at birth, but at two and four years of follow-up, it was found that the BMI z-score was inversely associated with DHA intra-erythrocyte levels during the 36 th week of gestation [60]. Consistent with this finding, other trials found an association between DHA levels in human milk and BMI in childhood up to age seven. No significant correlation between breast milk n-6/n-3 PUFAs’ ratio and body composition emerged [61]. 

Until recent times, LCPUFAs were not included in standard formula milk, which contained just their precursors (alpha linolenic acid and linoleic acid). During infancy, LCPUFAs levels inside red blood cells gradually reduce if the child is fed by standard formula milk, because of a limited capacity to synthesize ARA and DHA. This relative deficiency, which can limit the beneficial neurodevelopmental effect of LCPUFAs in the first years of life, as described in many studies, can be overcome with formula milk supplemented with adequate doses of LCPUFAs. For these reasons, currently, many formulas are enriched with LCPUFAs in quantities comparable with breast milk [62]. Even if the beneficial role of LCPUFAs on neuronal development is commonly accepted, it is still unclear if LCPUFA supplementation in preterm and term infants gives real short-term and long-term benefits. 

A recent meta-analysis including 11 randomized studies (3644 children globally) evaluated how n-3 LCPUFA supplementation during pregnancy could improve biometric parameters (BMI, body weight, fat mass) in the offspring. From this review, LCPUFAs seem not capable of preventing childhood obesity, but a lack of effect cannot be excluded due to the heterogeneity in the study design [63]. A Cochrane review of 15 randomized controlled trials (N = 1889) evaluated the safety and effectiveness of formula milk enriched with LCPUFAs in enhancing neurodevelopment, visual function, and physical growth in full-term babies. Among the numerous studies examined, which all had the limit of a small sample size, none showed a difference in biometric parameters until nine years old [64]. Similar conclusions were reported by Cochrane results from a review of studies conducted on preterm infants [65].

Many studies were conducted to assess whether LCPUFA supplementation and health parameters also in older children, but they were heterogeneous according to fatty acid dosage, lipid composition, and duration of supplementation. The Groningen randomized controlled trial studied how LCPUFA supplementation in the first two months of postnatal life could help the child’s growth: no benefits in biometric parameters (weight, height, BMI, head circumference) were found at 18 months and at nine years old; overweight/obesity prevalence rates and blood pressure values did not differ in the two intervention groups [66,67].

The wheezing illnesses study Leidsche Rijn, conducted on 2468 newborn babies, studied if there was a difference in cardiovascular profile in standard formula-fed infants compared to LCPUFA-enriched formula-fed infants. The results showed that carotid intimal media thickness, carotid distension, and blood pressure did not differ in the two groups at five years old [68]. 

Although the long-term impact is controversial, it is now recommended to assure adequate dietary n-3 LCPUFA intake in expectant mothers for its well-recognized neurocognitive benefits for the future newborn. Discussing its potential impact on adiposity, insulin sensitivity, and the other components of MetS through wider, prospective, and long-term clinical trials remains an important topic of investigation, especially for the implications in preventing children illness. 

## 6. Epigenetic Mechanisms of n-3 LCPUFAs Possibly Involved in MetS Pathways

Recent studies showed that epigenetic mechanisms may play a role in a wide range of diseases and also for metabolic syndrome initiation. Epigenetics investigates heritable modifications to the genome, which are independent of the DNA sequence [69]. This recent branch of research deals with heritable changes that modify the activity of genes, independently of the nucleotide coding (genotype), determining different phenotypes [70]. Given its dynamic nature, epigenetics has been suggestively described as the “interface between the genome and the environment” (Feil and Fraga, 2012). The period during which epigenetic activity is most effective lasts from conception to the second anniversary, underlying the crucial concept of “the first 1000 days” [71]. These chemical changes are vital for normal development, cellular processes, and cell-specific gene expression. Epigenetic modifications can be mediated by three main processes: non-coding RNAs, histone modifications, and DNA methylation [72,73,74].

### 6.1. Non-Coding RNA/miRNA

Non-coding RNAs (ncRNAs) are RNA molecules transcribed from DNA, but not translated into proteins. Epigenetic-related ncRNAs include miRNA, siRNA, piRNA, and lncRNA. In general, ncRNAs’ function is to regulate gene expression at the transcriptional and post-transcriptional level. Among ncRNAs, microRNAs (miRNAs) are currently the most studied. 

miRNAs are short non-coding RNAs composed of 22–23 nucleotides that are biologically highly conserved and that can modulate gene expression in response to different external factors [75]. Hundreds of miRNAs and thousands of targets involved in different cell processes (development, differentiation, proliferation, and apoptosis pathways) have been recognized in the human genome. 

It is recently emerging that the pathogenesis of many diseases can be explained by deregulated miRNAs [76]. 

miRNAs regulate various biological processes, but are mainly studied for their involvement in tumor genesis pathways, opening new perspectives for diagnosis and therapeutic strategies in the oncologic field.

Studies about the interaction between PUFAs and miRNAs are limited and focused primarily on tumor cell biology. miRNAs seem to be molecular targets of n-3 LCPUFAs for future cancer therapy, as emerges from the promising results of in vitro studies in colorectal cancer stem-like cells [77] and estrogen receptor-positive breast cancer cells [78]. In this perspective, n-3 LCPUFAs could represent a promising anti-tumor strategy with high efficacy and low toxicity. 

One study focused on the potential interaction between LCPUFAs and miRNAs in adipogenesis. MicroRNA-143 (miR-143) seems to stimulate adipocytes’ differentiation and is correlated with obese condition in mice fed with a high-fat diet. The authors, using chromatin immunoprecipitation, identified that the PPARγ signaling pathway promotes transcriptional activation of miR-143 in adipocytes [79]. 

The expression of miR-143 and PPARγ is increased by DHA, suggesting a potential role in adipose tissue differentiation [80].

Another in vitro experiment used a monocyte/macrophage cell line and an endothelial cell line to evaluate whether PUFAs affect miRNAs’ expression. The two cell lines were supplemented with PUFAs in a bioactive concentration (sufficient to reach a stable incorporation into the plasma membrane) and then exposed to an inflammatory environment. Results clearly showed that PUFAs could modulate miRNAs’ expression of both cell types independently of the presence/absence of an inflammatory trigger. Moreover, some miRNAs already correlated to vascular inflammation were found to be affected by cellular PUFA enrichment. These data suggest that vascular inflammation can be influenced by PUFA through miRNAs synthesized by cells involved in the inflammatory process [81] (Table 3).

### 6.2. Histone Acetylation

Chromatin is the ensemble of chromosomal DNA with proteins in the cellular nucleus. DNA in chromatin is enveloped around histone proteins, in units called ‘’nucleosomes’’. A nucleosome has 147 bp of DNA associated with an octameric core of histone proteins, consisting of two H3-H4 histone dimers surrounded by two H2A-H2B dimers. H1 histone associates with the linker DNA located between the nucleosomes. 

Histones’ post-translation modifications can occur in many ways, including ubiquitination, phosphorylation, acetylation, and methylation. Of the many described histone modification mechanisms is histone acetylation at the ε-amino group of lysine residues in the H3 and H4 tails, being one of the most-studied epigenetic mechanisms and seeming to promote transcription [82].

Currently, one animal experiment evaluated DHA epigenetic impact on histone acetylation, possibly correlated with MetS pathways. This work reported that n-3 fatty acids have a beneficial effect on dyslipidemia-induced epigenetic changes in maternal placenta and fetal liver of weanling female Wistar rats fed with control and different high-fat content diets (high-fat lard or high-fat fish oil). Maternal dyslipidemia caused a 4.75-fold increase in fetal liver triacylglycerol accumulation with a 78% decrease in the DNA-binding ability of PPAR-α. The combination EPA + DHA had a profound effect on modulating DNA methylation and histone acetylation in placenta and fetal liver [83].

### 6.3. DNA Methylation

DNA methylation is the most-studied epigenetic mechanism, which consists of the transferring of a methyl group to the cytosine C5 position, which results in the formation of 5-methylcytosine [84]. The modification of DNA methylation varies depending on nutrition and age; it seems to influence the gene expression pathways of several CV risk factors (atherosclerosis, inflammation) and MetS features such as hypertension, dyslipidemia, and diabetes [85,86,87,88]. 

In adult humans, differences in DNA methylation have been identified between people with high and low n-3 PUFA intakes. The Yup’ik have a traditional diet rich in fish, which is changing with Westernization, allowing for stratification of this population into high and low PUFA consumption. Increased DNA methylation with high PUFAs consumption was observed in 78% of the significant associations, with genomic instability occurring with hypomethylation: the increased methylation observed may, therefore, be beneficial to the stability of the genome [89]. In a separate cohort of Greek pre-adolescents (*n* = 69) was found an association between dietary fats and DNA methylation, including sites linked to inflammation as Peroxisome Proliferator-Activated Receptor (PPARα), Nuclear Factor kappa B (NFκB), Leptin (LEP), and interleukin [90].

DNA methylation in newborns from women who were given n-3 FA supplementation during late pregnancy has been evaluated in a few clinical trials [91]. DNA methylation changes following six-week supplementation with 3 g of n-3 FAs supplementation in 36 overweight and obese subjects showed that 308 CpG sites involved in inflammatory and immune response, type 2 diabetes, lipid metabolism, and cardiovascular signaling were differentially methylated after the intervention [92].

A double-blind randomized placebo-controlled trial conducted in pregnant women, randomized to receive either 400 mg of algal DHA daily or placebo from Weeks 18–22 of gestation until delivery, showed that supplementation induced a significant increase of *IGF2* Differentially-Methylated Regions (DMRs) in cord blood of infants of overweight mothers and a significant hypomethylation of H19 DMRs in infants of normal-weight mother [93]. Hypomethylation at *IGF2* DMRs has been previously proposed as one mechanism linking low birth weight, a high risk of diabetes, hypertension, and other metabolic diseases.

In another large double-blind randomized placebo-controlled trial, pregnant mothers consumed a daily dose of 800 mg DHA or placebo from 20 weeks of gestation to delivery. No difference in DNA methylation levels was found between the DHA and control group either at birth or at five years, but differences in 21 DMRs were found at birth, some of which persisted until five years. DMRs identified were located in genes involved in lipid exchange between membranes (*ESYT3*), appetite regulation (*CCK*), plasma membrane function (*SLC12A6*), immune function (*RAET1L* and *LTB*), and neurodevelopment/brain function (*SLC12A6, TRAK1, LPHN3*, and *RFPL2*). Results highlighted a major impact in males, suggesting that they may be more susceptible to prenatal exposure to DHA [94]. Recent literature is focusing on the long-term impact of DHA and EPA on the modulation of epigenetic determining a “metabolic risk”, but the significance of these changes still remain to be determined [95]. LCPUFAs’ mechanisms in modulation of DMR regions are synthesized in Table 4.

## 7. Materials and Methods

Inclusion criteria were: type of article (meta-analysis, review, systematic review, observational study, case-control study, longitudinal/prospective study, retrospective study, randomized controlled trial), publication date (May 2005–December 2018), species (both human and animal), and in vitro studies. Priority was given to data from clinical trials in children; then, if not available, to clinical trials in adults and ultimately preclinical studies. 

The literature research was performed on the MEDLINE^®^ and Cochrane database using terms individually or the Boolean ANDs and ORs. In the search strategy, the following terms were included: “pediatric obesity”, “metabolic syndrome”, “LCPUFA”, “fatty acids”, “dyslipidemia”, “insulin resistance”, “hypertension”, pregnancy”, “DHA supplementation”, “epigenetic”. All sources were retrieved between 2005 and 2019. 

## 8. Conclusions

Understanding through nutrigenetic research how diet affects human health, especially in the early phases of life, could be crucial to personalize nutritional intervention in order to prevent the transgenerational risk of obesity and its complications in children. n-3-LCPUFAs are well known for their health benefits in adults, but the beneficial effect of supplementation in children is still debated. The healthy properties associated mainly with DHA might be explained by epigenetic changes, even if the mechanisms involved are not clearly defined. Recent literature suggests that n-3 LCPUFA supplementation from diet may impact gene expression by epigenetic changes. This may influence inflammatory and other metabolic pathways and could be a key step to the initiation of metabolic syndrome, giving a chance to treat the disease with more specific strategies. Given the intrinsic limitations of pediatric clinical trials, due to the small population and gaps in follow-up time, further studies are needed to provide more answers about n-3 LCPUFAs’ influence on gene expression and modulation, particularly regarding the benefits for infants and their future health. 

## Figures and Tables

**Table 1:** Main n-3 LCPUFAs.

Common Name	Lipid Number	Systematic Nomenclature
α-Linolenic Acid (ALA)	18:3 (n-3)	*all*-*cis*-9,12,15-octadecatrienoic acid
Stearidonic Acid (SDA)	18:4 (n-3)	*all*-*cis*-6,9,12,15-octadecatetraenoic acid
Eicosatetraenoic Acid (ETA)	20:4 (n-3)	*all-cis*-8,11,14,17-eicosatetraenoic acid
Eicosapentaenoic Acid (EPA)	20:5 (n-3)	*all*-*cis*-5,8,11,14,17-eicosapentaenoic acid
Docosapentaenoic acid (DPA)	22:5 (n-3)	*all-cis*-7,10,13,16,19-docosapentaenoic acid
Docosahexaenoic Acid (DHA)	22:6 (n-3)	*all-cis*-4,7,10,13,16,19-docosahexaenoic acid

**Table 2:** Main n-6 LCPUFAs.

Common Name	Lipid Number	Systematic Nomenclature
Linoleic Acid (LA)	18:2 (n−6)	*all-cis*-9,12-octadecadienoic acid
γ -Linolenic Acid (GLA)	18:3 (n−6)	*all-cis*-6,9,12-octadecatrienoic acid
Dihomo-γ-Linolenic Acid (DGLA)	20:3 (n−6)	*all-cis*-8,11,14-eicosatrienoic acid
Arachidonic Acid (AA, ARA)	20:4 (n−6)	*all-cis*-5,8,11,14-eicosatetraenoic acid
Adrenic acid (ADA)	22:4 (n−6)	*all-cis*-7,10,13,16-docosatetraenoic acid

**Table 3:** LCPUFAs’ impact on miRNA-mediated epigenetic processes.

Biological Process	Epigenetic Target	Mechanism	Effect
Adipogenesis	miRNA-143 target of PPARγ in adipocyte differentiation stimulator	increase miRNA-143	regulation of adipogenesis and obesity
Vascular inflammation	miRNA-21, miRNA-125a, mi-RNA146a, miR-146b, miRNA-155 targets of TLR signaling	decrease miRNAs	modulator of vascular inflammation

**Table 4:** LCPUFAs’ impact on DMR regions in human controlled trials.

Population	Genes Involved	Biological Function
Greek adolescents with different fat dietary intake [89]	*NCOA1*, *PDE3A*	susceptibility to diet-induced obesity, leptin pathway
Newborns from DHA-supplemented pregnant women in Mexico [92]	*IGF2* *P3* *H19*	fetal growth, development, and metabolism
Children of DHA-supplemented pregnant women (at birth and at 5 years) [93]	*ESYT3*, *SLC12A6*, *CCK*, *RAET1L*, *LTB*	lipid membrane exchange, plasma membrane function, appetite regulation, immune function, neurodevelopment
DHA-supplemented overweight and obese adults in Canada [91]	*AKT3*, *PTEN*, *BAX*, etc. *IGFBP5*, *KLK6*, etc. *PRKAG2*, *PRKCZ*, *HDAC4*, *PRKAG2*	immune response lipid metabolism type 2 diabetes CV signaling

## Abbreviations

LCPUFAs	Long Chain Polyunsaturated Fatty Acids
MetS	Metabolic Syndrome
FAs	Fatty Acids
WHO	World Health Organization
DHA	Docosahexaenoic Acid
EPA	Eicosapentaenoic Acid
ALA	Alpha Linolenic Acid
CVD	Cardiovascular Diseases
LDL	Low-Density Lipoprotein
HDL	High-Density Lipoprotein
RCT	Randomized Controlled Trials
PPAR	Peroxisome Proliferator Activated Receptor
BMI	Body Mass Index
HOMA-IR	Homeostatic Model Assessment of Insulin Resistance
DMRs	Differentially-Methylated Regions
NcRNAs	Non-coding RNAs

## References

[1] Ogden C.L., Carroll M.D. (2012). Prevalence of obesity and trends in body mass index among US children and adolescents. JAMA.

[2] Ogden C.L., Flegal K.M. (2002). Prevalence and trends in overweight among US children and adolescents. JAMA.

[3] Craig M., Hales M.D. (2017). Prevalence of Obesity Among Adults and Youth: United States. NCHS Data Brief..

[4] Seema K., Aaron S.K. (2017). Review of Childhood Obesity: From Epidemiology, Etiology, and Comorbidities to Clinical Assessment and Treatment. Mayo Clinic Proceedings.

[5] WHO European Childhood Obesity Surveillance Initiative: Overweight and Obesity among 6–9-year-old Children. Report of the Third Round of Data Collection 2012–2013. http://www.euro.who.int/en/health-topics/disease-prevention/nutrition/publications/2018/who-european-childhood-obesity-surveillance-initiative-overweight-and-obesity-among-69-year-old-children.-report-of-the-third-round-of-data-collection-20122013-2018.

[6] Spinelli A., Nardone P. (2016). Centro nazionale per la prevenzione delle malattie e la promozione della salute, Cnapps-Iss. OKkio alla Salute: I dati nazionali. http://www.epicentro.iss.it/okkioallasalute/dati2016.asp.

[7] Valerio G., Licenziati M.R. (2014). Health consequences of obesity in children and adolescents. Minerva Pediatr..

[8] Fruh S.M. (2017). Obesity: Risk factors, complications, and strategies for sustainable long-term weight management. J. Am. Assoc. Nurse Pract..

[9] Mottillo S., Filion K.B. (2010). The metabolic syndrome and cardiovascular risk a systematic review and meta-analysis. J. Am. Coll. Cardiol..

[10] Valerio G., Maffeis C. (2018). Diagnosis, treatment and prevention of pediatric obesity: Consensus position statement of the Italian Society for Pediatric Endocrinology and Diabetology and the Italian Society of Pediatrics. Ital. J. Pediatr..

[11] Desai M., Jellyman J.K. (2015). Epigenomics, gestational programming and risk of metabolic syndrome. Int. J. Obes..

[12] Abedi E., Sahari M.A. (2014). Long-chain polyunsaturated fatty acid sources and evaluation of their nutritional and functional properties. Food Sci. Nutr..

[13] Strobel C., Jahreis G. (2012). Survey of n-3 and n-6 polyunsaturated fatty acids in fish and fish products. Lipids Health Dis..

[14] Cheng J., Ma Y. (2017). Accumulation and health risks of heavy metals in the seafood from China. J. Hyg. Res..

[15] Stratakis N., Roumeliotaki T. (2017). Fish and seafood consumption during pregnancy and the risk of asthma and allergic rhinitis in childhood: A pooled analysis of 18 European and US birth cohorts. Int. J. Epidemiol..

[16] Khozin-Goldberg I., Iskandarov U. (2011). LC-PUFA from photosynthetic microalgae: Occurrence, biosynthesis, and prospects in biotechnology. Appl. Microbiol. Biotechnol..

[17] Oehlenschläger J. (2012). Seafood: Nutritional Benefits and Risk Aspects. Int. J. Vitam. Nutr. Res..

[18] Wang C., Harris W.S. (2006). N-3 Fatty acids from fish or fish-oil supplements, but not alpha-linolenic acid, benefit cardiovascular disease outcomes in primary- and secondary-prevention studies: A systematic review. Am. J. Clin. Nutr..

[19] Saini R.K., Keum Y.S. (2018). Omega-3 and omega-6 polyunsaturated fatty acids: Dietary sources, metabolism, and significance—A review. Life Sci..

[20] Wilber C.G., Levine V.E. (1950). Fat metabolism in Alaskan Eskimos. Experimantal Med. Surg..

[21] Figueiredo P.S., Inada A.C. (2017). Fatty Acids Consumption: The Role Metabolic Aspects Involved in Obesity and Its Associated Disorders. Nutrients.

[22] Alberti K.G., Zimmet P. (2006). Metabolic syndrome—A new world-wide definition. A Consensus Statement from the International Diabetes Federation. Diabet Med..

[23] May A.L., Kuklina E.V. (2012). Prevalence of cardiovascular disease risk factors among US adolescents. Pediatrics.

[24] Zimmet P., Alberti K.G. (2007). The metabolic syndrome in children and adolescents - an IDF consensus report. Pediatr. Diabetes.

[25] Huang P.L. (2009). A comprehensive definition for metabolic syndrome. Dis. Model Mech..

[26] Grundy S.M., Cleeman J.I. (2005). Diagnosis and management of the metabolic syndrome. Exec. Summ. Cardiol. Rev..

[27] Eslick G.D., Howe P. (2009). RBenefits of fish oil supplementation in hyperlipidemia: A systematic review and meta-analysis. Int. J. Cardiol..

[28] Mozaffarian D., Wu J.H. (2011). Omega-3 fatty acids and cardiovascular disease: Effects on risk factors, molecular pathways, and clinical events. J. Am. Coll. Cardiol..

[29] Lopez-Huertas E. (2012). The effect of EPA and DHA on metabolic syndrome patients: A systematic review of randomised controlled trials. Br. J. Nutr..

[30] Shearer G.C., Savinova O.V. (2012). Fish oil -- how does it reduce plasma triglycerides?. Biochim. Biophys. Acta.

[31] Bradberry J.C., Hilleman D.E. (2013). “Overview of omega-3 Fatty Acid therapies”. J. Formul. Manag..

[32] Miller P.E., Van Elswyk M. (2014). Long-chain omega-3 fatty acids eicosapentaenoic acid and docosahexaenoic acid and blood pressure: A meta-analysis of randomized controlled trials. Am. J. Hypertens..

[33] Colussi G., Catena C. (2017). Impact of omega-3 polyunsaturated fatty acids on vascular function and blood pressure: Relevance for cardiovascular outcomes. Nutr. Metab. Cardiovasc. Dis..

[34] Pase M.P., Grima N.A. (2011). Do long-chain n-3 fatty acids reduce arterial stiffness? A meta-analysis of randomised controlled trials. Br. J. Nutr..

[35] Mozaffarian D., Geelen A. (2005). Effect of fish oil on heart rate in humans: A meta-analysis of randomized controlled trials. Circulation.

[36] Mori T.A. (2014). Dietary n-3 PUFA and CVD: A review of the evidence. Proc. Nutr. Soc..

[37] Balakumar P., Taneja G. (2012). Fish oil and vascular endothelial protection: Bench to bedside. Free Radic Biol. Med..

[38] Chen C., Yu X. (2015). Effects of Omega-3 Fatty Acid Supplementation on Glucose Control and Lipid Levels in Type 2 Diabetes: A Meta-Analysis. PLoS ONE.

[39] Wu J.H., Micha R. (2012). Omega-3 fatty acids and incident type 2 diabetes: A systematic review and meta-analysis. Br. J. Nutr..

[40] Scaglioni S., Verduci E. (2006). PPAR-2 Pro12Ala Variant, Insulin Resistance and Plasma Long-chain Polyunsaturated Fatty Acids in Childhood Obesity. Pediatric Res..

[41] Monsalve F.A., Pyarasani R.D. (2013). Peroxisome proliferator-activated receptor targets for the treatment of metabolic diseases. Mediators Inflamm..

[42] Itoh T., Murota I. (2006). Synthesis of docosahexaenoic acid derivatives designed as novel PPARgamma agonists and antidiabetic agents. Bioorg. Med. Chem..

[43] Bender N., Portmann M. (2014). Fish or n3-PUFA intake and body composition: A systematic review and meta-analysis. Obes. Rev..

[44] Burrows T., Collins C.E. (2011). Omega-3 index, obesity and insulin resistance in children. Int. J. Pediatr. Obes..

[45] López-Alarcón M., Inda-Icaza P. (2018). A randomized control trial of the impact of LCPUFA-ω3 supplementation on body weight and insulin resistance in pubertal children with obesity. Pediatr. Obes..

[46] Svensson V., Johansson E. (2018). Omega-3 fatty acids does not affect physical activity and body weight in primary school children - a double-blind randomized placebo-controlled trial. Sci. Rep..

[47] Nobili V., Bedogni G. (2011). Docosahexaenoic acid supplementation decreases liver fat content in children with non-alcoholic fatty liver disease: Double-blind randomised controlled clinical trial. Arch. Dis. Child..

[48] Juárez-López C., Klünder-Klünder M. (2013). Omega-3 polyunsaturated fatty acids reduce insulin resistance and triglycerides in obese children and adolescents. Pediatr. Diabetes..

[49] López-Alarcón M., Martínez-Coronado A. (2011). Supplementation of n3 long-chain polyunsaturated fatty acid synergistically decreases insulin resistance with weight loss of obese prepubertal and pubertal children. Arch. Med. Res..

[50] De Ferranti S.D., Milliren C.E. (2014). Using high-dose omega-3 fatty acid supplements to lower triglyceride levels in 10- to 19-year-olds. Clin. Pediatr..

[51] Mejía-Barradas C.M., Del-Río-Navarro B.E. (2014). The consumption of n-3 polyunsaturated fatty acids differentially modulates gene expression of peroxisome proliferator-activated receptor alpha and gamma and hypoxia-inducible factor 1 alpha in subcutaneous adipose tissue of obese adolescents. Endocrine.

[52] Berger J.P., Akiyama T.E. (2005). PPARs: Therapeutic targets for metabolic disease. Trends Pharmacol. Sci..

[53] Tyagi S., Gupta P. (2011). The peroxisome proliferator-activated receptor: A family of nuclear receptors role in various diseases. J. Adv. Pharm. Technol. Res..

[54] Ross M.G., Desai M. (2013). Developmental programming of offspring obesity, adipogenesis, and appetite. Clin. Obstet Gynecol..

[55] Carlson S.E., Colombo J. (2016). Docosahexaenoic Acid and Arachidonic Acid Nutrition in Early Development. Adv. Pediatr..

[56] Lauritzen L., Brambilla P. (2016). DHA Effects in Brain Development and Function. Nutrients.

[57] Carlson S.E., Colombo J. (2013). DHA supplementation and pregnancy outcomes. Am. J. Clin. Nutr..

[58] Woo Baidal J.A., Locks L.M. (2016). Risk Factors for Childhood Obesity in the First 1000 Days: A Systematic Review. Am. J. Prev. Med..

[59] Wood K., Mantzioris E. (2018). The effect of maternal DHA supplementation on body fat mass in children at 7 years: Follow-up of the DOMInO randomized controlled trial. Prostaglandins Leukot Essent Fatty Acids..

[60] Foster B.A., Escaname E. (2017). Randomized Controlled Trial of DHA Supplementation during Pregnancy: Child Adiposity Outcomes. Nutrients.

[61] Pedersen L., Lauritzen L. (2012). Polyunsaturated fatty acid content of mother’s milk is associated with childhood body composition. Pediatric Res..

[62] Koletzko B., Lien E. (2008). The roles of long-chain polyunsaturated fatty acids in pregnancy, lactation and infancy: Review of current knowledge and consensus recommendations. J. Perinat. Med..

[63] Vahdaninia M., Mackenzie H. (2018). The effectiveness of ω-3 polyunsaturated fatty acid interventions during pregnancy on obesity measures in the offspring: An up-to-date systematic review and meta-analysis. Eur. J. Nutr..

[64] Simmer K., Patole S.K. (2011). Longchain polyunsaturated fatty acid supplementation in infants born at term. Cochrane Database Syst. Rev..

[65] Schulzke S.M., Patole S.K. (2011). Long-chain polyunsaturated fatty acid supplementation in preterm infants. Cochrane Database Syst. Rev..

[66] Rosenfeld E., Beyerlein A. (2009). IPD meta-analysis shows no effect of LC-PUFA supplementation on infant growth at 18 months. Acta Paediatr..

[67] De Jong C., Boehm G. (2011). The Groningen LCPUFA study: No effect of short-term postnatal long-chain polyunsaturated fatty acids in healthy term infants on cardiovascular and anthropometric development at 9 years. Pediatr Res..

[68] Pluymen L., Dalmeijer G.W. (2018). Long-chain polyunsaturated fatty acids in infant formula and cardiovascular markers in childhood. Matern Child Nutr..

[69] Felsenfeld G. (2014). A brief history of epigenetics. Cold Spring Harb. Perspect Biol..

[70] Trerotola M., Relli V. (2015). Epigenetic inheritance and the missing heritability. Hum Genomics..

[71] Bhutta Z.A., Das J.K. (2013). Evidence-based interventions for improvement of maternal and child nutrition: What can be done and at what cost?. Lancet.

[72] Morales S., Monzo M. (2017). Epigenetic regulation mechanisms of microRNA expression. Biomol. Concepts..

[73] Jones M.J., Goodman S.J. (2015). DNA methylation and healthy human aging. Aging Cell.

[74] Gräff J., Tsai L.H. (2013). Histone acetylation: Molecular mnemonics on the chromatin. Nat. Rev. Neurosci..

[75] Valinezhad Orang A., Safaralizadeh R. (2014). Mechanisms of miRNA-Mediated Gene Regulation from Common Downregulation to mRNA-Specific Upregulation. Int. J. Genomics..

[76] Ha T.Y. (2011). MicroRNAs in Human Diseases: From Cancer to Cardiovascular Disease. Immune Netw..

[77] Sam M.R., Tavakoli-Mehr M. (2018). Omega-3 fatty acid DHA modulates p53, survivin, and microRNA-16-1 expression in KRAS-mutant colorectal cancer stem-like cells. Genes Nutr..

[78] LeMay-Nedjelski L., Mason-Ennis J.K. (2018). Omega-3 Polyunsaturated Fatty Acids Time-Dependently Reduce Cell Viability and Oncogenic MicroRNA-21 Expression in Estrogen Receptor-Positive Breast Cancer Cells (MCF-7). Int. J. Mol. Sci..

[79] Bae I.S., Park P.J. (2017). PPARγ-mediated G-protein coupled receptor 120 signaling pathway promotes transcriptional activation of miR-143 in adipocytes. Gene.

[80] McGregor R.A., Choi M.S. (2011). MicroRNAs in the regulation of adipogenesis and obesity. Curr. Mol. Med..

[81] Roessler C., Kuhlmann K. (2017). Impact of Polyunsaturated Fatty Acids on miRNA Profiles of Monocytes/Macrophages and Endothelial Cells-A Pilot Study. Int. J. Mol. Sci..

[82] Handy D.E., Castro R. (2011). Epigenetic modifications: Basic mechanisms and role in cardiovascular disease. Circulation.

[83] Ramaiyan B., Talahalli R.R. (2018). Dietary Unsaturated Fatty Acids Modulate Maternal Dyslipidemia-Induced DNA Methylation and Histone Acetylation in Placenta and Fetal Liver in Rats. Lipids.

[84] Moore L.D., Le T. (2013). DNA methylation and its basic function. Neuropsychopharmacology.

[85] Wise I.A., Charchar F.J. (2016). Epigenetic Modifications in Essential Hypertension. Int. J. Mol. Sci..

[86] Zhong J., Agha G. (2016). The Role of DNA Methylation in Cardiovascular Risk and Disease: Methodological Aspects, Study Design, and Data Analysis for Epidemiological Studies. Circ. Res..

[87] Braun K.V., Voortman T. (2016). The role of DNA methylation in dyslipidaemia: A systematic review. Prog. Lipid Res..

[88] Davegårdh C., García-Calzón S. (2018). DNA methylation in the pathogenesis of type 2 diabetes in humans. Mol. Metab..

[89] Aslibekyan S., Wiener H.W. (2014). DNA methylation patterns are associated with n-3 fatty acid intake in Yup’ik people. J. Nutr..

[90] Voisin S., Almén M.S. (2015). Dietary fat quality impacts genome-wide DNA methylation patterns in a cross-sectional study of Greek preadolescents. Eur. J. Hum. Genet..

[91] Heijmans B.T., Tobi E.W. (2008). Persistent epigenetic differences associated with prenatal exposure to famine in humans. Proc. Natl. Acad. Sci. USA.

[92] Tremblay B.L., Guénard F. (2017). Epigenetic changes in blood leukocytes following an omega-3 fatty acid supplementation. Clin. Epigenet..

[93] Lee H.S., Barraza-Villarreal A. (2014). Dietary supplementation with polyunsaturated fatty acid during pregnancy modulates DNA methylation at IGF2/H19 imprinted genes and growth of infants. Physiol. Genom..

[94] Van Dijk S.J., Zhou J. (2016). Effect of prenatal DHA supplementation on the infant epigenome: Results from a randomized controlled trial. Clin. Epigenet..

[95] Herrera B.M., Keildson S. (2011). Genetics and epigenetics of obesity. Maturitas.

